# Global, regional and national burdens of otitis media in children and adolescents from 1990 to 2021 and its predictions to 2040

**DOI:** 10.3389/fpubh.2025.1552405

**Published:** 2025-07-03

**Authors:** Ru Chen, Jing Deng, Yao Sun, Dongxun Sun, Haibin Lu, Xinfang Jiao, Feng Zhu, Liangjie Lu, Guoqi Sima

**Affiliations:** Department of Otolaryngology, The First Hospital of Jiaxing, The Affiliated Hospital of Jiaxing University, Jiaxing, China

**Keywords:** otitis media, children and adolescents, global burden of disease, incidence, DALYs

## Abstract

**Background:**

Otitis media (OM), including acute OM, chronic OM, and OM with effusion, is associated with varying degrees of hearing impairment. Children and adolescents (CAAs) are particularly vulnerable to OM. However, epidemiological data on OM in CAAs is relatively scarce. This study investigates the global, regional, and national burden of OM in CAAs from 1990 to 2021, with projections extending to 2040.

**Methods:**

Data were extracted from Global Burden of Diseases (GBD) 2021 on incidence, prevalence, deaths and disability-adjusted life years (DALYs). Trends were evaluated using the metric of estimated annual percentage change (EAPC). Subgroup analyses were conducted according to socio-demographic index (SDI), and age. Additionally, projections were estimated for 2040 using the Nordpred model.

**Results:**

Globally, the rates with their 95% uncertainty intervals (UI) in 2021 were 12473.66 (7287.91–19931.88) for incidence, 2438.73 (1918.03–3055.21) for prevalence, 0.0095 (0.0022–0.0320) for deaths, and 49.33 (27.68–78.84) for DALYs. From 1990 to 2021, the EAPC and its 95% UI of incidence rate increased by 0.13 (0.11–0.16), while EAPC of deaths −3.79 (−4.07 to −3.52), prevalence −0.08 (−0.09 to −0.07), and DALYs −0.2 (−0.23 to −0.17) decreased. The aforementioned indicators are negatively correlated with the SDI. Regionally, both mortality rates and DALYs significantly decrease with increasing SDI. Sub-Saharan Africa and South Asia have high levels of incidence and prevalence. At the national level, countries with a high burden of OM are primarily concentrated in Sub-Saharan Africa and South Asia. For example: Pakistan, India, Ethiopia, Kenya, Nepal, Bangladesh, Somalia, South Sudan, Mozambique, Burundi, and Madagascar. From 2022 to 2040, the incidence rate, prevalence, and DALYs of OM are expected to show a downward trend. However, the mortality rate may slightly increase.

**Conclusion:**

From 1990 to 2021, there has been some progress in the management of OM. However, the incidence in CAAs has increased. Epidemiological data vary across different regions and countries, with regions and countries with lower SDI typically experiencing a heavier burden. It is necessary to implement dynamic monitoring of OM in CAAs and develop strategies to mitigate the future burden of this disease.

## Introduction

Otitis media (OM) is a common inflammatory disease of the middle ear, influenced by multiple factors such as viral or bacterial infections, eustachian tube dysfunction, environmental factors, genetics, and allergies (1). In addition to the bothersome clinical symptoms, one of the major complications that OM imposes on individuals is hearing loss (2). OM includes acute OM, chronic OM, and OM with effusion (OME), each associated with different risks and patterns of hearing impairment. Acute OM is often caused by bacterial or viral infections and typically resolves on its own or with antibiotic treatment when indicated, and the associated hearing loss is usually mild and short-term (3). Chronic OM, characterized by recurrent infections, tympanic membrane perforations (4), often leads to more severe and progressive hearing loss. OME, defined by the presence of middle ear fluid without acute infection, can be categorized as either acute or chronic based on its duration. Acute OME is typically self-limiting, and the associated hearing loss is usually mild and transient. In contrast, chronic OME—characterized by prolonged effusion and low resolution rates—may lead to persistent mild-to-moderate conductive hearing loss (5). Most forms of OM—except for chronic cases—are self-limiting or effectively managed with appropriate treatment, and therefore typically do not result in permanent hearing impairment. However, when hearing loss does occur and becomes persistent, its impact can be substantial. The harmful effects of hearing loss include affecting language development in children, increasing the risk of cognitive impairment and dementia in the older adult, causing anxiety and depression in patients, and raising unemployment rates (6). A study conducted in the United States indicated that individuals with hearing loss had a significantly higher likelihood of unemployment compared to those with normal hearing (odds ratio [OR]: 2.20) (7). These consequences impose a significant burden on society.

As one of the leading causes of hearing loss, OM imposes varying burdens across different populations and regions. In terms of population, children are the most vulnerable group to OM, followed by young adults (8). According to the United Nations definition, “child” refers to individuals aged 0–9 years, and “adolescents” refers to those aged 10–19 years, CAAs make up 30% of the global population and account for about 90% of OM incidence (9, 10). Therefore, CAAs are a significant component of the OM burden. Regionally, the imbalance is reflected in the fact that the burden of OM may be heavier in some underdeveloped areas and low SDI regions (10). The global burden of disease (GBD) 2021 collaborators on upper respiratory tract infections and OM have shown that the incidence, mortality, and DALYs of OM have improved compared to 1990 (8). A similar conclusion was drawn in a study by Jin et al. (10). Although the burden of OM has improved since 1990, the absolute numbers remain high: it was reported that in 2021, approximately 391 million people were affected by OM (8). Additionally, these studies considered OM across all age groups as a whole, leading to a lack of specific epidemiological data for OM in CAAs. Understanding the global, regional, and national trends of OM in this vulnerable group could be helpful for policy development and adjustments.

The GBD database provides a framework for analyzing the trends of OM across different age groups, time periods, and regions. This study aims to analyze the impact of region, SDI, age, and other factors on the incidence, prevalence, mortality, and DALYs of OM from 1990 to 2021 using the publicly available data from the GBD study.

## Methods

### Overview

GBD 2021 provides comprehensive data on multiple diseases at the global level, across 21 regions, and for 204 countries, covering epidemiological information on OM from 1990 to 2021. This study primarily analyzes the disease burden of OM in CAAs, CAAs here are defined as individuals aged 0–19 years. In the GBD 2021 study, OM was characterized as a middle ear infection, encompassing acute OM (AOM), chronic OM (COM), and hearing loss resulting from COM within the nonfatal outcome models. OM is categorized under ICD-10 codes H65 through H75.83 and ICD-9 codes 381 to 384.9. This study assessed the burden of OM by examining incidence, prevalence, and deaths cases and rates, as well as disability-adjusted life years (DALYs) and their respective rates.

### Data sources and estimation framework

The data is derived from the retrieval function of the Global Health Data Exchange. The study set various detailed parameters, including “causes of death or injury,” “incidence, prevalence, deaths, and DALYs,” “number, rates,” “otitis media,” “SDI, 21 regions, and 204 countries,” “0–19 years,” “male, female, both,” and others. The methods for data collection, processing, and analysis in the GBD study have been comprehensively detailed in prior publications (11–13).

In this study, incidence refers to the number of newly diagnosed OM cases within a specific time period, while prevalence represents the total number of individuals with OM during the same period. DALYs indicate the overall health burden of OM, encompassing years lived with disability (YLDs) and years of life lost (YLLs) due to premature death.

GBD 2021 utilizes advanced modeling methodologies to assess the burden of OM. The estimation of incidence and prevalence is primarily based on DisMod-MR 2.1 (a Bayesian meta-regression disease modeling tool), which synthesizes various disease-related parameters, epidemiological patterns, and spatial data to produce reliable OM burden estimates. GBD estimates mortality using the Cause of Death Ensemble Model, a robust statistical approach that integrates multiple predictive models to generate cause-specific mortality rates. DALYs represent a combined measure of YLDs and YLLs. All data presented in the tables and figures of this study were derived from GBD-related models and can be accessed in the GBD Results database. For a more detailed explanation of the calculation methods, please refer to the following reference (14).

### Socio-demographic index

The socio-demographic index (SDI) is a key composite indicator of development status in the GBD. It integrates various factors such as per capita income, average educational attainment, and fertility rates. The SDI ranges from 0 to 1, with higher values indicating higher levels of development. In 2021, countries and regions were divided into five groups based on the SDI quintiles: Low SDI (0 ≤ SDI < 0.466), Low-middle SDI (0.466 ≤ SDI < 0.619), Middle SDI (0.619 ≤ SDI < 0.712), High-middle SDI (0.712 ≤ SDI < 0.810), and High SDI (0.810 ≤ SDI ≤ 1.000). A complete list of SDI values and quintile classifications for all regions and countries is provided in Supplementary Table S1.

### Statistical analysis

In 2021, a comprehensive assessment was conducted to measure the burden of OM, including its age-standardized incidence rate (ASIR), age-standardized prevalence rate (ASPR), age-standardized death rate (ASDR), DALYs rate and numbers. Using the aforementioned indicators as a baseline, trend charts of OM changes from 1990 to 2021 were generated at the global level, across the five SDI regions. Additionally, the study investigated how sociodemographic factors shape OM’s effects, examining variations in disease burden across different age groups. Using a log-linear regression model, the estimated annual percentage change (EAPC) was calculated for each indicator to reflect trends in the data. To conduct an inequality analysis based on DALYs, it is recommended to use the Slope Index of Inequality (SII) and the Concentration Index to assess absolute and relative income inequalities between countries (15). By comparing the slopes of the SII for the years 1990 and 2021, the improvement in the imbalance of OM can be demonstrated—the flatter the slope, the more evenly the disease is distributed across the country. The Concentration Index ranges from −1 to 1, with values close to 0 indicating better balance. Positive and negative values signify that the disease burden is more concentrated in higher and lower SDI regions, respectively. The decomposition analysis categorized DALYs based on three factors—aging, population, and epidemiological—to reveal how each factor contributes to health inequalities and the health burden. Frontier analysis identifies potential health improvements attainable given the current developmental level in 204 countries. Building on the timeframes adopted in previous high-quality studies (16–18), we considered key factors such as public health strategy (which typically focuses on long-term disease improvement), data accuracy, and the interpretative value of projections (where shorter timeframes may provide limited insights, while longer ones may reduce accuracy). The Nordpred forecasting model was employed to predict OM in CAAs up to the year 2040. The Nordpred model integrates features of the age–period–cohort framework, breaking down the time dimension into age, period, and cohort effects, and has been optimized and refined to more accurately project future disease trends.

## Results

### Global burden of OM

**Figure 1:**
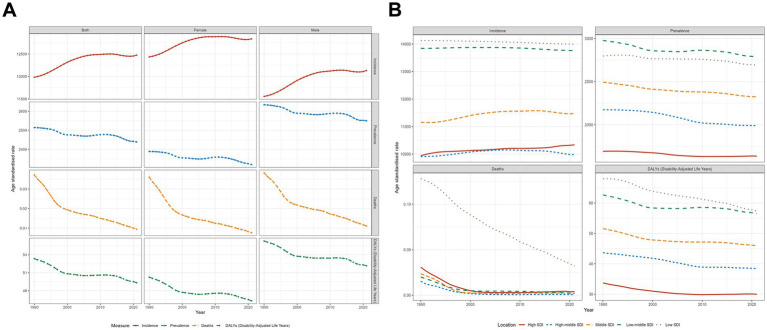
Trends in otitis media incidence, prevalence, deaths, and disability-adjusted life-years from 1990 to 2021. **(A)** Global trends stratified by sex (both sexes, females, and males), **(B)** Trends across five socio-demographic index (SDI) levels.

In 2021, the number of new OM cases in CAAs was 316 million (95% UI 184 to 507), with an ASIR of 12473.66 (95% UI 7287.91 to 19931.88) per 100,000. The prevalence number of OM patients in CAAs was 64 million (95% UI 50 to 80), with an ASPR of 2438.73 (95% UI 1918.03 to 3055.21) per 100,000. The number of deaths among OM patients in CAAs was 233 (95% UI 54 to 792), with an ASDR of 0.0095 (95% UI 0.0022 to 0.0320) per 100,000. The number of DALYs among OM patients in CAAs was 1.29 million (95% UI 0.73 to 2.07), with a rate of 49.33 (95% UI 27.68 to 78.84) per 100,000. From 1990 to 2021, the EAPC of ASIR, ASPR, ASDR, and DALY rates were 0.13 (0.11 to 0.16), −0.08 (−0.09 to −0.07), −3.79 (−4.07 to −3.52), and −0.2 (−0.23 to −0.17), respectively. For a more detailed description of the data from 1990 and 2021, please refer to Supplementary Tables S2, S3. Figure 1A illustrates the global trends in the data.

The analysis based on SDI subgroups revealed that the low-middle SDI region bears the highest burden of OM in terms of incidence, prevalence, and DALYs. Specifically, this region reported 100 million incidences (95% UI: 58–162), 21 million prevalent cases (95% UI: 17–26), and 430938.64 DALYs (95% UI: 240910.43–685915.39). In contrast, the low SDI region recorded the highest ASIR of 13992.70 (95% UI: 8185.72–22412.55) and the highest DALYs rate of 57.33 (95% UI: 32.08–90.31). Additionally, the low-middle SDI region had the highest ASPR at 2785.39 (95%UI: 2202.24–3483.80). Despite all regions having ASDR below 0.1, the low SDI region exhibited a significantly higher number of deaths, totaling 193.62 (95% UI: 32.89–709.94), compared to the other four regions. From 1990 to 2021, the EAPC of mortality rates in all regions showed a significant decline, with the highest decline in the high-middle SDI region, EAPC = −9.39 (95% UI: −11.23 to −7.52). Across all regions, the changes in incidence rates were relatively stable, while the declining trends in prevalence and DALYs were relatively gradual. Figure 1B illustrates the data trends across different SDI regions.

**Figure 2:**
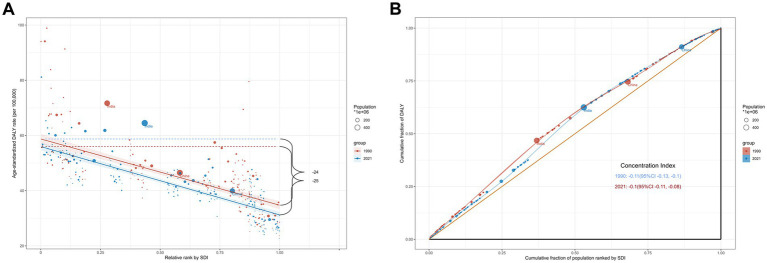
The inequality slope index **(A)** and concentration index **(B)** for disability-adjusted life-years of otitis media worldwide in 1990 and 2021.

In 1990 and 2021, the SII values for DALYs were −25 and −24, respectively, and the Concentration Index increased from −0.11 in 1990 to −0.10 in 2021 (Figure 2). The above data indicate that the health inequality burden of OM between high- and low-income countries has improved, but it remains a difficult issue to address.

Age-stratified subgroup analysis shows that children aged 0–5 bear the highest burden in ASIR, ASPR, and ASDR, while children aged 5–9 bear the highest burden in DALYs (Supplementary Figure S1).

### Regional burden of OM

The specific values for data of incidence, prevalence, mortality, DALYs, and EAPC across different regions are detailed in Supplementary Tables S2, S3. This study employs graphical representations to enhance data visualization.

**Figure 3:**
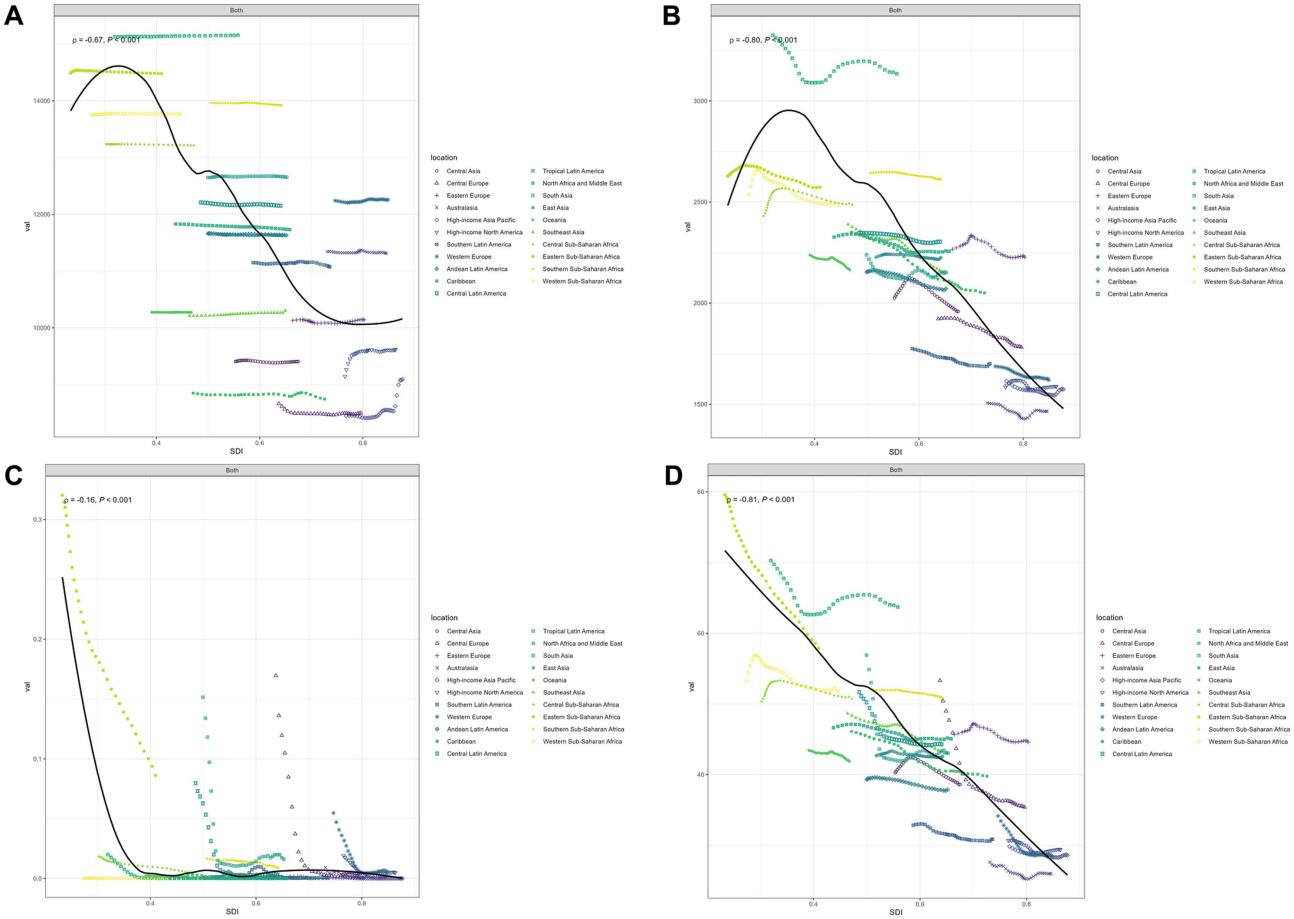
Age-standardized incidence **(A)**, age-standardized prevalence **(B)**, age-standardized deaths **(C)**, and age-standardized disability-adjusted life-years **(D)** for otitis media by SDI, 1990–2021. ‘Both’ refers to combined data for males and females.

Figure 3A displays the smoothed curve of OM incidence rates across 21 different regions. An R value of −0.67 and *p* < 0.001 indicate that the incidence of OM follows a trend of first increasing and then decreasing with the rise in SDI. In most regions, the incidence of OM remains relatively stable.

Figure 3B displays the smoothed curve of OM prevalence rates across 21 different regions. An R value of −0.80 and *p* < 0.001 indicate that the prevalence of OM follows a trend of first increasing and then decreasing with the rise in SDI. In most regions, the prevalence of OM remains relatively stable.

Figure 3C displays the smoothed curve of OM death rates across 21 different regions. An R value of −0.16 and *p* < 0.001 indicate that the mortality curve of OM shows a significant downward trend with the rise in SDI. The curve trends in different regions tend to stabilize or decline. The most significant downward trends are observed in five regions: Eastern Sub-Saharan Africa, Central Latin America, Tropical Latin America, Central Europe, and Western Europe.

Figure 3D displays the smoothed curve of OM DALYs rates across 21 different regions. An R value of −0.81 and *p* < 0.001 indicate that the DALYs curve of OM shows a significant downward trend with the rise in SDI. The most significant downward trends are observed in six regions: Eastern Sub-Saharan Africa, South Asia, Central Latin America, Tropical Latin America, Central Europe, and Western Europe.

### National burden of OM

**Figure 4:**
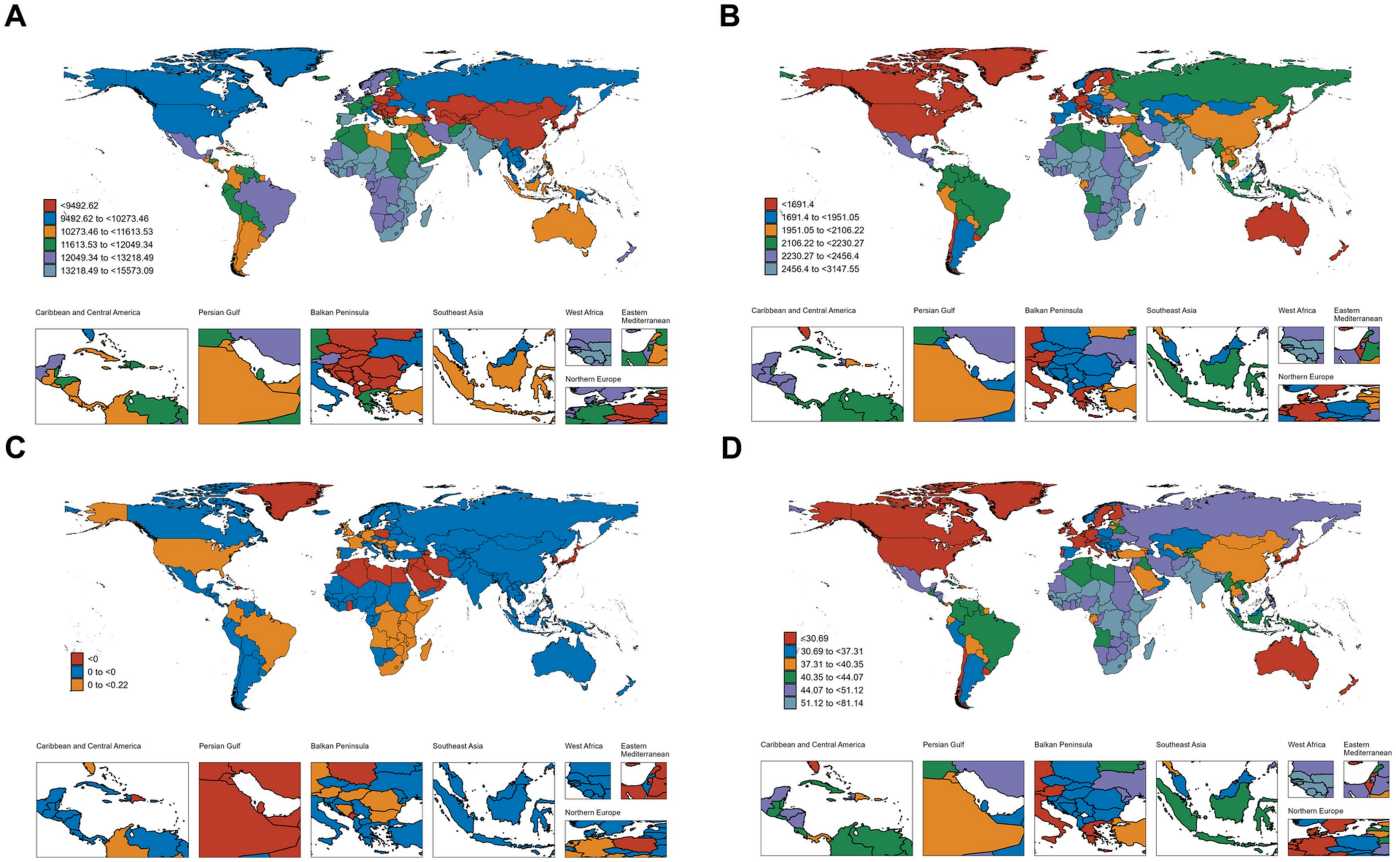
Geographical distribution of age-standardized incidence **(A)**, age-standardized prevalence **(B)**, age-standardized deaths **(C)**, and age-standardized disability-adjusted life-years **(D)**.

For ASIR, the top five countries and their respective values are: Pakistan 15573.09, Spain 15254.58, India 15172.83, Ethiopia 15170.83, and Kenya 15151.60. Taiwan (province of China), with an ASIR of 6474.71, has the lowest ranking. For ASPR, the top five countries and their respective values are: India 3147.55, Pakistan 3125.31, Nepal 3113.03, Bangladesh 3061.97, and Somalia 3028.77. Monaco, with a value of 1357.28, ranks the lowest in ASPR. For ASDR, the top five countries and their respective values are: Somalia 0.22, South Sudan 0.21, Mozambique 0.12, Burundi 0.10, and Madagascar 0.09. United Arab Emirates ranks the lowest in ASDR. For DALYs, the top five countries and their respective values are: Somalia 81.14, South Sudan 65.32, India 64.49, Nepal 62.86, and Burundi 62.73. Monaco, with a value of 22.66, ranks the lowest in DALYs. The visualization of the country burden for OM can be seen in Figure 4.

### Decomposition analysis based on DALYs

**Figure 5:**
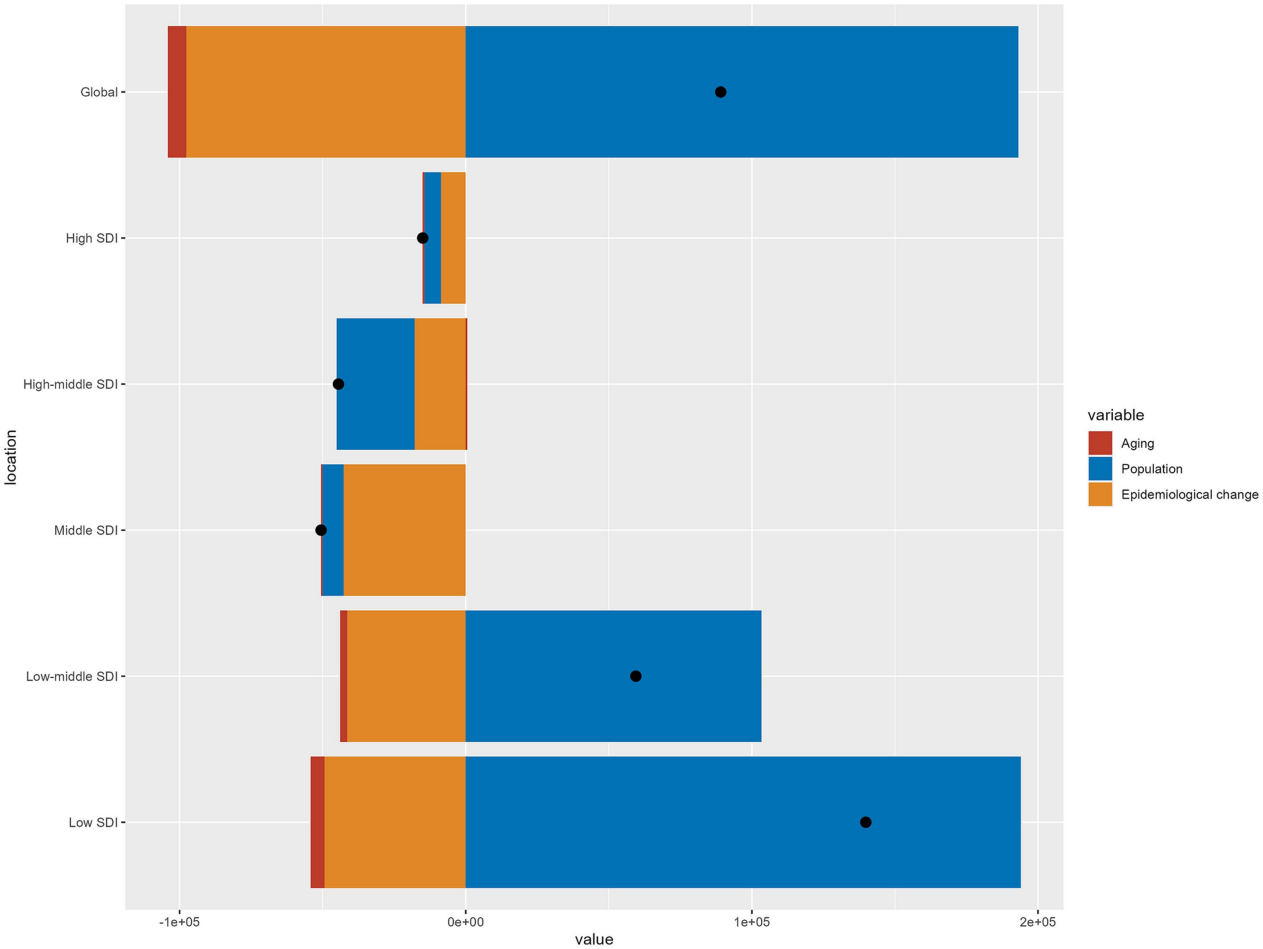
Changes in otitis media disability-adjusted life years according to population-level determinants of aging, population growth, and epidemiological change from 1990 to 2021 at the global level and by sociodemographic index quintile.

Figure 5 visually presents the decomposition analysis results for the global and different SDI regions. Globally, 216.80% of the variation in DALYs was attributed to changes in population, −7.15% to aging, and −109.65% to epidemiological changes. The proportion of aging in different SDI regions were as follows: low SDI (−3.47%), low-middle SDI (−4.33%), middle SDI (0.85%), high-middle SDI (−1.33%), and high SDI (3.85%). The proportion of population growth in different SDI regions were: low SDI (138.74%), low-middle SDI (173.90%), middle SDI (14.76%), high-middle SDI (61.15%), and high SDI (38.38%). The proportion of epidemiological changes in different SDI regions were: low SDI (−35.27%), low-middle SDI (−69.56%), middle SDI (84.39%), high-middle SDI (40.18%), and high SDI (57.77%).

### Frontier analysis based on DALYs

**Figure 6:**
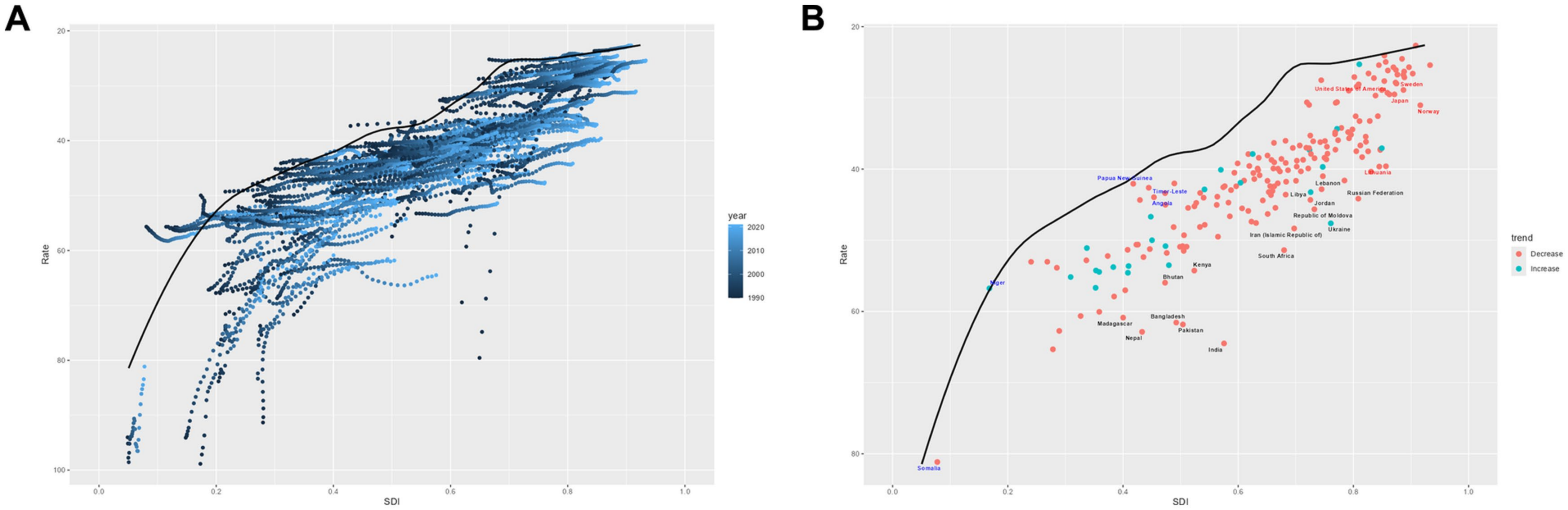
Frontier analysis on the basis of sociodemographic-index and age-standardized disability-adjusted life-years per 100,000 of otitis media from 1990 to 2021. **(A)** 1990–2021; **(B)** 2021.

The frontier analysis based on DALYs for 204 countries is shown in Figure 6. The trend depicted in Figure 6A shows that as the SDI increases, the disparities in disease burden among different countries are gradually decreasing. In Figure 6B, the 15 countries with the largest effective disparities are displayed in black font, indicating that these countries have the greatest potential for improving the burden of OM: Madagascar, Nepal, Bangladesh, Bhutan, Pakistan, India, Kenya, South Africa, Iran (Islamic Republic of), Libya, Republic of Moldova, Jordan, Lebanon, Ukraine, and Russian Federation. Frontier nations with low-SDI (<0.5) include Somalia, Niger, Papua New Guinea, Timor-Leste, and Angola. Countries in high SDI (>0.85) regions that still have significant potential for improvement include: United States of America, Sweden, Japan, Norway, and Lithuania.

### Projections of OM

**Figure 7:**
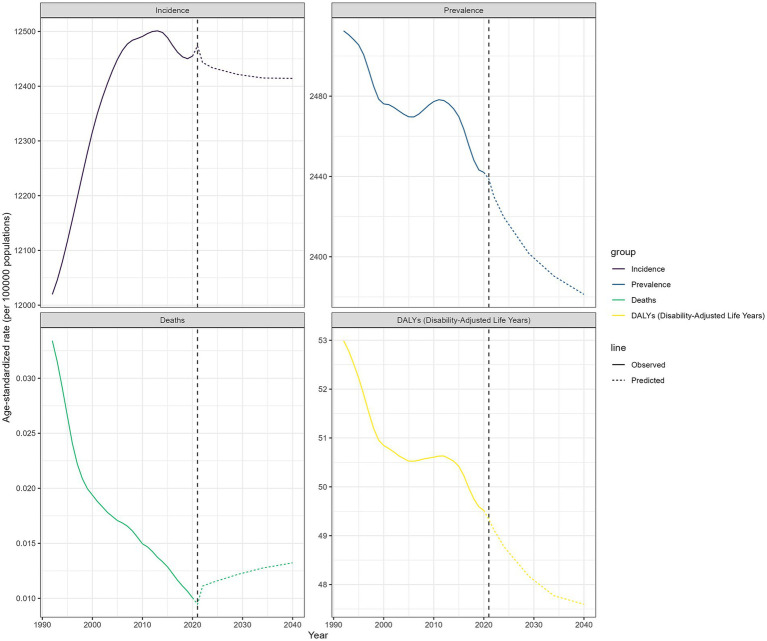
The age-standardized incidence, prevalence, deaths, and disability-adjusted life-years for global otitis media for the observational period (1990–2021) and the projection period (2022–2040).

This study predicts the trend of OM in CAAs from 2022 to 2040, with the forecast curve shown in Figure 7. Globally, by 2040, ASIR, ASPR, and DALYs are expected to decline, while ASDR may slightly increase. By 2040, the ASIR is expected to reach 12414.27, with ASPR, ASDR, and DALYs at 2381.26, 0.0132, and 47.60, respectively.

## Discussion

To our knowledge, this is the first study to evaluate the burden of OM among the susceptible population of CAAs at the global, regional, and national levels, adding detail to the currently limited epidemiological data on OM among CAAs. Over the past 30 years, globally, the mortality rate of OM within CAAs has significantly decreased, and both prevalence and DALYs have improved. However, the ASIR has increased. Overall, regions and countries with lower SDI bear a heavier burden of OM.

From 1990 to 2021, the global population grew from 5.2 billion to approximately 7.8 billion, and the newly added young population is a susceptible group for OM. Industrialization and economic development may have led to environmental degradation. Additionally, advancements in healthcare may lead to the detection of more hidden OM cases. These factors could contribute to the increased incidence of OM. Fortunately, the burden of OM in terms of prevalence, mortality, and DALYs has been decreasing. It is commendable that the number of deaths caused by OM and the mortality rate of OM have significantly decreased compared to data from 1990 (846 versus 233, EAPC = −3.79). The prevalence and DALYs of OM also show a downward trend (EAPC = −0.08 for prevalence and −0.2 for DALYs). The improvement in these data may be attributed to enhancements in medical services and technology, economic development, introduction and improvement of guidelines, and the promotion of OM vaccines, among other factors.

Our findings suggest that OM burden is generally negatively correlated with SDI, as indicated by subgroup analysis. However, research exploring the relationship between SDI classification and OM burden remains limited, likely because SDI is a broad classification, with each SDI group encompassing multiple countries or regions. To better understand this relationship, SDI can be examined through its key components—income level and fertility rate—as these factors have been theoretically linked to OM burden. Higher SDI regions generally have better healthcare infrastructure (19), enabling more standardized treatment and improved OM management. Additionally, fertility rate estimates suggest that some low-income and low-SDI regions exhibit higher fertility rates (13). Given that children are the primary susceptible population for OM, this, in turn, may be another key factor contributing to the higher OM burden observed in low-SDI and low-middle SDI regions.

It is worth noting that low SDI regions and Eastern Sub-Saharan Africa account for the vast majority of OM death cases (193.62 for low SDI regions, 201.56 for Eastern Sub-Saharan Africa, and 233.06 for global). This indicates that there is still room for improvement in these regions in the prevention and treatment of OM. Setting aside these regions, the global mortality rate of OM in CAAs has remained low (generally less than 0.1 per 100,000 in both 1990 and 2021), indicating that deaths account for a relatively small proportion of the overall burden of OM. However, the hearing loss caused by the disease has become a significant burden affecting DALYs. Previous studies have shown that the burden of OM varies significantly across countries and regions with different economic levels (20). As a comprehensive evaluation indicator, the SDI not only reflects socio-demographic development but also, to some extent, represents the economic status of a region. As the data show, across the majority of regions, increases in SDI generally correspond to reductions in DALYs. This indicates that social development has heightened the attention given to OM, particularly in the following six regions: Eastern Sub-Saharan Africa, South Asia, Central Latin America, Tropical Latin America, Central Europe, and Western Europe. Although DALYs have improved compared to the past, the overall trend remains relatively flat. To further explore the different factors influencing DALYs in OM, we decomposed the original DALYs into aging, population, and epidemiological changes. Globally, the primary factor influencing DALYs is population growth (accounting for 216.80%), followed by epidemiological changes (accounting for −109.65%), while aging has a relatively lower impact on DALYs (−7.15%). However, the contributions of these three factors vary significantly across different SDI levels. Overall, aging is minimally affected by SDI and generally accounts for the smallest proportion. In regions with low, low-middle, and high-middle SDI levels, population growth is the primary driver of DALYs. In contrast, in middle and high SDI regions, epidemiological factors play a more significant role.

For ASIR and ASPR, Sub-Saharan Africa and South Asia hold higher values, with representative countries including Pakistan, India, Ethiopia, Kenya, Nepal, Bangladesh, and Somalia. In Sub-Saharan Africa, delays in seeking medical treatment are very common and may lead to an increase in disease prevalence. Additionally, environmental factors such as poor ventilation and smoke exposure may increase the risk of OM incidence and prevalence (21). In middle- and low-income countries in Africa, the availability of evidence-based information and adherence to guidelines are insufficient (22). Furthermore, high bacterial resistance limits the treatment options for OM in Sub-Saharan Africa (23). Unfortunately, few studies have analyzed OM data from South Asia as a whole. Therefore, exploring the reasons behind the high OM burden in this region could benefit from examining countries with high ASIR and ASPR, such as Pakistan, India, Bangladesh, and Nepal. The significant OM burden in these countries may be associated with factors such as poverty, lack of education, malnutrition, poor immunity, inadequate sanitation, and insufficient healthcare coverage (24–29). These data indicate that Sub-Saharan Africa and South Asia bear a heavier burden of OM. Some representative countries in these regions have shortcomings in OM prevention and treatment, highlighting the need for timely adjustments in strategies to address the high burden of OM. As the only European country ranked in the top five for ASIR, Spain’s high incidence rate may be attributed to factors such as a lack of breastfeeding, exposure to secondhand smoke, and the level of parental engagement in seeking medical care when their children are ill (30–32).

Overall, the higher burden of OM in these regions and countries may be attributed to a combination of factors, including limited awareness of medical care, poor hygiene conditions, smoke exposure, low adherence to clinical guidelines, antibiotic resistance, low education levels, and childhood malnutrition. To address these challenges, several feasible interventions are recommended. First, community-based health promotion programs—locally adapted education and prevention initiatives conducted through clinics, schools, and community organizations—can help raise awareness of OM risk factors, promote early recognition of symptoms, and encourage timely healthcare-seeking behaviors. Second, integrating OM education into school curricula and community outreach initiatives can empower individuals with the necessary knowledge to seek early intervention and adopt preventive measures. Third, strengthening local diagnostic capabilities by enhancing training for healthcare professionals and improving access to affordable diagnostic tools is critical for early detection and timely management of the condition. Finally, these strategies should be integrated within broader public health policies that address underlying socio-economic determinants, thereby creating a more sustainable and comprehensive approach to reducing the OM burden in high-risk regions. In addition, improving basic sanitation infrastructure and promoting smoke-free environments—particularly through indoor air quality interventions—may help reduce OM risk in vulnerable communities. Addressing antibiotic resistance through stewardship programs and ensuring nutritional support for children, especially in regions with high rates of malnutrition, should also be part of an integrated strategy to reduce the overall burden of OM.

To better understand the potential improvements in DALYs across different countries, a frontier analysis was conducted using DALYs and SDI data from the 1990–2021 GBD study. The distance between each point and the frontier line represents the disparity between a country’s DALYs and the ideal condition, which theoretically can be reduced or eliminated through dedicated efforts. Through this frontier analysis, 15 countries with the highest potential for improvement were identified. Additionally, the study explored the potential for improvement among countries in different SDI regions. Notably, in regions with an SDI below 0.5, Papua New Guinea, Timor-Leste, and Angola have achieved relatively outstanding OM management under limited conditions, serving as exemplars. However, some high SDI countries exhibit OM burdens that are inconsistent with their development status, including the United States of America, Sweden, Japan, Norway, and Lithuania. This discrepancy may be due to stricter diagnostic criteria for OM, more rigorous antibiotic usage standards, and other factors (5, 33). Therefore, the OM governance experiences of these exemplary countries should be explored and adopted. Furthermore, it is essential to investigate the underlying causes of high DALYs in certain countries, as this may contribute to further reducing the OM burden.

Using data from GBD 2021, this study also projected the burden of OM in CAAs for the period 2022–2040. Over the next 20 years, ASIR, ASPR, and DALYs rates are expected to show a downward trend. However, the ASDR may slightly increase. Regarding the potential increase in OM mortality, the following factors are speculated: the gradual rise in bacterial resistance may reduce the effectiveness of antibiotic treatments for OM, and the improvement of diagnostic and testing technologies in underdeveloped regions may lead to the identification of previously undiagnosed severe OM cases. Although the predictive model indicated a slight increase in mortality, the absolute number and proportion of deaths remain at relatively low levels. These predictions indicate that the burden of OM in CAAs will see sustained improvement in the future. Although progress has been made in the prevention and treatment of OM over the past 30 years (33–35), there remains room for improvement in specific countries and regions. Enhancing public education, further refining and promoting clinical guidelines, advancing vaccine development, tackling antibiotic-resistant pathogens, and controlling risk factors may contribute to further reducing the burden of OM. These efforts are worthwhile and deserve our collective commitment.

Our study has several limitations. Firstly, The OM data in the GBD study are derived from estimates using statistical models. Discrepancies may exist between OM data obtained from family reports and physician-based diagnoses. In underdeveloped regions, misdiagnosis and underdiagnosis of OM may occur, leading to an underestimation of the OM burden in some areas. Secondly, The GBD database treats OM as a single entity, despite the significant differences in the burden caused by different types of OM. For instance, AOM, when treated appropriately, typically has a limited impact on patient burden, whereas chronic and secretory OM may significantly affect hearing, quality of life, and social burden. A more detailed classification of OM in the GBD database in the future could help address this issue. Furthermore, For OM, where mortality rates are already low, using incidence, prevalence, and DALYs as metrics may not fully capture the burden. Factors such as patients’ psychological health, quality of life, and family economic costs also have a significant impact. However, there is a lack of effective methods to quantify the burden of these indicators. We hope that future epidemiological studies on OM can address these shortcomings and further validate our findings.

In conclusion, over the past 30 years, significant progress has been made in the prevention and treatment of OM, as evidenced by notable improvements in mortality rates, and decreases in prevalence and DALYs. However, the ASIR has increased, and significant regional differences still exist. The burden of OM is negatively correlated with the SDI. The ASIR and ASPR remain high in Sub-Saharan Africa, South Asia, and some representative countries in these regions, calling for greater attention to OM-related health issues in these areas. Predictions indicate that the future OM burden will further decrease. It is necessary to implement dynamic monitoring of OM in CAAs and develop strategies to mitigate the future burden of this disease.

## Data Availability

The original contributions presented in the study are included in the article/Supplementary material, further inquiries can be directed to the corresponding author.
