# Supplementary figures and images for: Global, regional and national burdens of otitis media in children and adolescents from 1990 to 2021 and its predictions to 2040
Source: Front Public Health. 2025 Jul 3;13:1552405. doi: 10.3389/fpubh.2025.1552405 (PMC12267207; doi:10.3389/fpubh.2025.1552405)

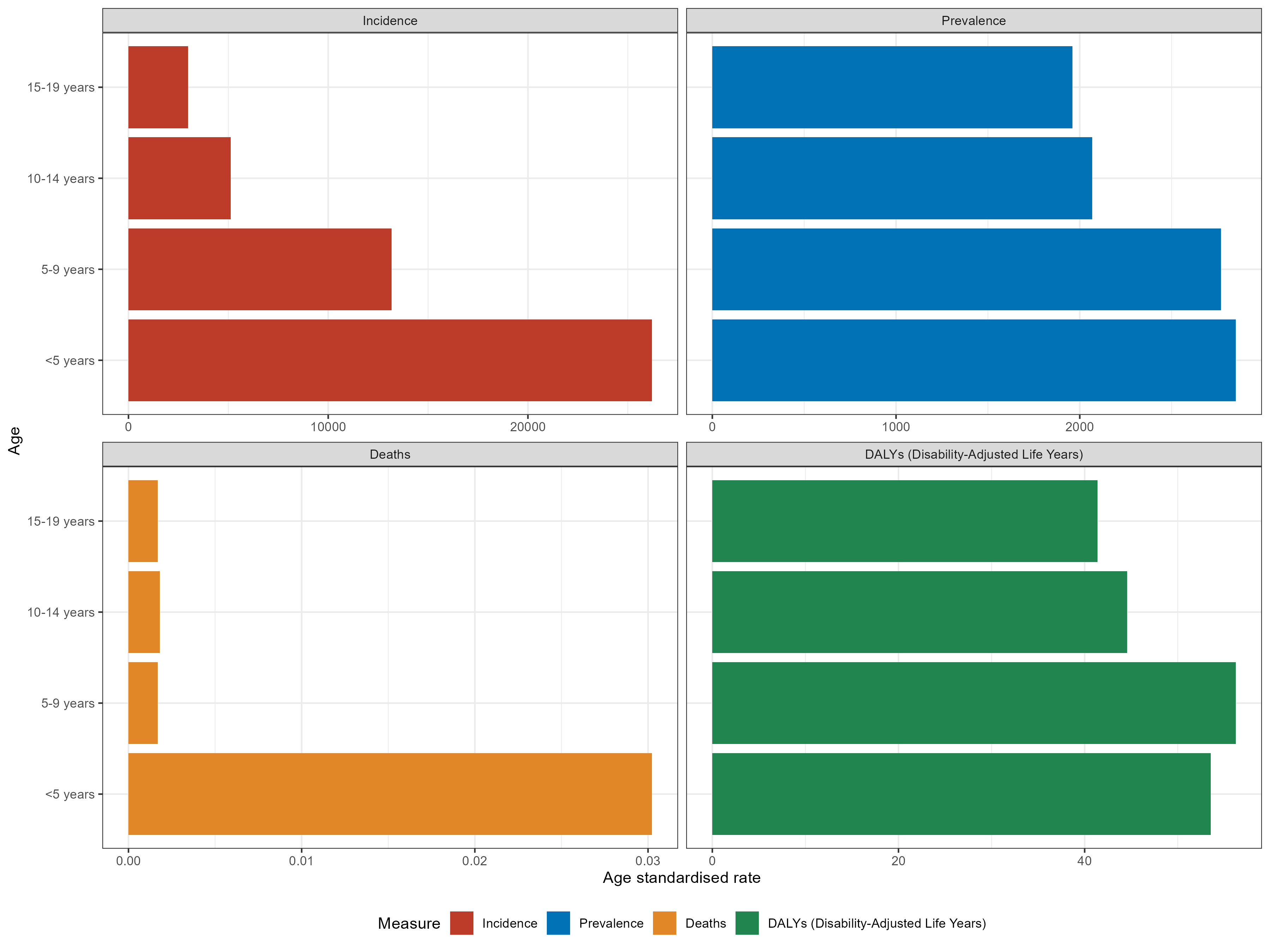

Supplement: Supplementary file 1 [file Image_1.TIFF]
