# Supplementary material for: Global, regional and national burdens of otitis media in children and adolescents from 1990 to 2021 and its predictions to 2040
Source: Front Public Health. 2025 Jul 3;13:1552405. doi: 10.3389/fpubh.2025.1552405 (PMC12267207; doi:10.3389/fpubh.2025.1552405)
Supplement: Supplementary file 3 [file Table_2.DOCX]

| Table S2. Incidence, prevalence, deaths, DALYs of otitis media in 2021 at the global and regional levels | | | | | | |  |  |
| --- | --- | --- | --- | --- | --- | --- | --- | --- |
| Location (2021) | Incidence cases (95% UI) | ASR (95% UI) | Prevalence cases (95% UI) | ASR (95% UI) | Deaths cases (95% UI) | ASR (95% UI) | DALYs cases (95% UI) | ASR (95% UI) |
| Global | 315840504.56 (183669259.16-506624007.31) | 12473.66 (7287.91-19931.88) | 63786655.91 (50083894.09-79985543.00) | 2438.73 (1918.03-3055.21) | 233.06 (54.28-791.51) | < 0.1 | 1294002.00 (725868.35-2067509.75) | 49.33 (27.68-78.84) |
| High SDI | 21993499.52 (14566037.23-32625478.20) | 10336.61 (6888.66-15250.63) | 3667476.40 (2886857.50-4572192.99) | 1635.19 (1294.11-2031.56) | 7.84 (5.44-9.59) | < 0.1 | 67759.12 (37626.05-110990.16) | 29.98 (16.64-49.19) |
| High-middle SDI | 28494248.00 (16326089.44-46128051.53) | 9978.07 (5776.28-16017.30) | 5953138.04 (4626699.70-7474813.35) | 1988.23 (1552.27-2489.67) | 1.69 (1.06-2.37) | < 0.1 | 115798.99 (64486.63-188104.68) | 38.45 (21.42-62.49) |
| Middle SDI | 80809222.11 (46137741.98-131037505.69) | 11478.63 (6606.70-18492.58) | 17201870.35 (13364093.81-21628102.97) | 2324.30 (1812.64-2915.47) | 14.06 (9.28-19.06) | < 0.1 | 342640.62 (191754.56-552074.74) | 46.04 (25.79-74.20) |
| Low-middle SDI | 100684246.44 (57857185.80-161751830.34) | 13761.23 (7937.64-22037.30) | 21143153.11 (16691498.66-26462960.54) | 2785.39 (2202.24-3483.80) | 15.65 (3.85-56.04) | < 0.1 | 430938.64 (240910.43-685915.39) | 56.56 (31.64-90.06) |
| Low SDI | 83647728.73 (49034443.26-133774103.03) | 13992.70 (8185.72-22412.55) | 15779136.80 (12420344.78-19834173.85) | 2690.96 (2116.76-3383.58) | 193.62 (32.89-709.94) | < 0.1 | 336030.43 (188020.20-529210.68) | 57.33 (32.08-90.31) |
| Andean Latin America | 2687515.84 (1575550.68-4337693.75) | 11629.27 (6832.88-18745.48) | 487030.55 (379961.82-615840.05) | 2071.14 (1617.93-2617.40) | 0.11 (0.05-0.21) | < 0.1 | 8929.10 (4919.98-14890.81) | 37.88 (20.87-63.18) |
| Australasia | 801557.37 (460307.47-1272117.96) | 11313.78 (6547.45-17861.58) | 106876.79 (79769.07-140804.59) | 1465.20 (1097.15-1926.57) | 0.07 (0.04-0.12) | < 0.1 | 1908.94 (1001.05-3227.70) | 26.01 (13.63-44.00) |
| Caribbean | 1704023.23 (992233.77-2724180.25) | 11625.36 (6786.82-18548.39) | 336285.92 (265099.27-421284.77) | 2225.32 (1757.91-2785.51) | 0.15 (0.05-0.47) | < 0.1 | 6459.61 (3598.52-10518.97) | 42.59 (23.73-69.38) |
| Central Asia | 3371864.74 (1914131.81-5493254.25) | 9406.90 (5333.24-15344.93) | 684947.41 (539619.48-859840.22) | 1958.41 (1537.82-2463.43) | 0.06 (0.02-0.12) | < 0.1 | 13472.95 (7513.40-22103.22) | 38.61 (21.51-63.35) |
| Central Europe | 1875294.98 (1140004.90-2921667.60) | 8496.83 (5181.80-13197.46) | 411440.15 (320245.11-518448.95) | 1781.31 (1395.28-2233.94) | 0.76 (0.45-1.11) | < 0.1 | 8207.36 (4541.52-13506.37) | 35.40 (19.63-58.26) |
| Central Latin America | 9634076.13 (5540604.01-15444661.81) | 12151.23 (7038.17-19375.31) | 1931585.68 (1514401.23-2408545.32) | 2303.39 (1812.30-2867.05) | 2.68 (1.64-3.83) | < 0.1 | 37408.23 (21167.44-61166.10) | 44.30 (25.05-72.49) |
| Central Sub-Saharan Africa | 10065265.58 (5907765.76-16078570.92) | 13212.93 (7742.15-21129.52) | 1844555.70 (1437091.74-2322503.64) | 2487.47 (1935.12-3134.86) | 3.84 (0.35-19.40) | < 0.1 | 37537.91 (21062.93-60592.87) | 50.75 (28.45-81.93) |
| East Asia | 28795825.17 (16282867.87-46902515.92) | 8744.38 (4997.44-14121.41) | 7048313.27 (5498682.49-8818235.41) | 2050.62 (1604.94-2561.88) | 0.16 (0.05-0.37) | < 0.1 | 137470.56 (76787.99-223628.05) | 39.80 (22.24-64.82) |
| Eastern Europe | 4436393.68 (2473666.10-7295915.15) | 10139.32 (5711.88-16531.37） | 1024080.28 (807117.12-1273692.17) | 2226.80 (1762.47-2763.55) | 0.08 (0.05-0.12) | < 0.1 | 20634.99 (11680.68-33223.35) | 44.61 (25.25-71.87) |
| Eastern Sub-Saharan Africa | 33480778.03 (19416590.32-52914661.67) | 14477.99 (8382.78-22906.57) | 5877641.84 (4558321.91-7491089.73) | 2573.42 (1994.38-3281.23) | 201.56 (33.76-745.38) | 0.09 (0.01-0.32) | 132459.27 (70729.59-223205.72) | 57.89 (30.94-97.45) |
| High-income Asia Pacific | 2461548.19 (1082541.01-3012814.71) | 9091.21 (5358.66-14380.74) | 461147.15 (354679.14-586328.11) | 1572.94 (1219.08-1991.78) | 0.0007 (0.0004-0.0012) | < 0.1 | 8488.82 (4626.30-14154.21) | 28.59 (15.59-47.72) |
| High-income North America | 7720867.13 (5780552.44-9973522.32) | 9619.58 (7212.59-12408.13) | 1359722.76 (1110648.36-1638208.00) | 1587.68 (1305.71-1902.86) | 3.82 (2.60-4.76) | < 0.1 | 25380.77 (14265.73-41901.92) | 29.36 (16.50-48.60) |
| North Africa and Middle East | 27228866.43 (15901990.74-43965105.41) | 11730.65 (6881.75-18889.24) | 5317682.71 (4164746.10-6690321.69) | 2256.08 (1768.10-2838.06) | 0.03 (0.0052-0.0979) | < 0.1 | 106460.54 (59546.38-170750.45) | 45.07 (25.21-72.31) |
| Oceania | 691751.05 (407811.49-1115596.72) | 10272.01 (6016.79-16651.36) | 139406.65 (108985.78-175076.98) | 2164.07 (1685.38-2723.50) | 0.11 (0.01-0.73) | < 0.1 | 2684.70 (1492.85-4365.83) | 41.87 (23.27-68.06) |
| South Asia | 94982310.59 (54111891.33-152890654.92) | 15155.72 (8696.83-24248.57) | 21162082.49 (16639485.06-26493402.30) | 3134.21 (2472.75-3917.93) | 0.23 (0.03-0.73) | < 0.1 | 433490.18 (241815.54-688114.63) | 63.72 (35.59-101.23) |
| Southeast Asia | 22412589.63 (12706903.30-36235475.92) | 10302.31 (5864.52-16584.43) | 4879983.36 (3747069.07-6188591.57) | 2156.85 (1663.83-2727.04) | 1.15 (0.46-2.18) | < 0.1 | 97860.81 (54411.30-159530.36) | 43.10 (24.00-70.27) |
| Southern Latin America | 1943739.50 (1108357.40-3086151.39) | 11075.33 (6403.43-17434.95) | 318090.74 (243251.69-407549.63) | 1700.10 (1306.9-2172.94) | 0.083 (0.038-0.143) | < 0.1 | 5841.38 (3200.84-9711.75) | 30.87 (16.90-51.50) |
| Southern Sub-Saharan Africa | 4240379.68 (2433091.82-6827885.75) | 13919.39 (8012.96-22361.67) | 812359.27 (634689.21-1027787.72) | 2610.34 (2040.95-3301.89) | 2.76 (1.23-6.12) | < 0.1 | 15829.06 (8849.45-25545.86) | 50.71 (28.33-81.90) |
| Tropical Latin America | 8162391.29 (4738538.65-13015938.84) | 12654.65 (7354.47-20165.00) | 1414490.53 (1088704.50-1812343.71) | 2152.06 (1660.12-2752.52) | 10.63 (7.26-14.24) | < 0.1 | 28345.81 (16133.78-45924.20) | 43.04 (24.51-69.69) |
| Western Europe | 10282563.12 (6119295.57-16317481.71) | 12256.12 (7364.00-19309.69) | 1422359.38 (1067826.35-1854671.87) | 1619.17 (1220.99-2105.99) | 4.50 (3.04-5.75) | < 0.1 | 25129.88 (13761.98-42023.43) | 28.40 (15.57-47.57) |
| Western Sub-Saharan Africa | 38860903.21 (22800642.46-62137474.32) | 13762.45 (8037.68-22074.43) | 6746573.29 (5299991.69-8504655.11) | 2486.77 (1951.91-3135.96) | 0.26 (0.03-0.91) | < 0.1 | 140001.14 (79054.89-222040.15) | 51.86 (29.28-82.17) |

DALYs, disability-adjusted life years; ASR, age-standardized rate; SDI, socio-demographic index.
