# Supplementary material for: Global, regional and national burdens of otitis media in children and adolescents from 1990 to 2021 and its predictions to 2040
Source: Front Public Health. 2025 Jul 3;13:1552405. doi: 10.3389/fpubh.2025.1552405 (PMC12267207; doi:10.3389/fpubh.2025.1552405)
Supplement: Supplementary file 4 [file Table_3.DOCX]

| Table S3. EAPC of ASIR, ASPR, ASDR, and DALYs rates from 1990 to 2021 | | | | |
| --- | --- | --- | --- | --- |
| Location (1990-2021) | EAPC: ASIR | EAPC: ASPR | EAPC: ASDR | EAPC: DALYs |
| Global | 0.13 (0.11-0.16) | -0.08 (-0.09 to -0.07) | -3.79 (-4.07 to -3.52) | -0.2 (-0.23 to -0.17) |
| High SDI | 0.09 (0.09-0.10) | -0.15 (-0.17 to -0.13) | -6.04 (-7.75 to -4.29) | -0.35 (-0.42 to -0.28) |
| High-middle SDI | 0.04 (0.02-0.07) | -0.36 (-0.39 to -0.33) | -9.39 (-11.23 to -7.52) | -0.46 (-0.50 to -0.42) |
| Middle SDI | 0.12 (0.09-0.15) | -0.21 (-0.23 to -0.20) | -6.62 (-8.54 to -4.65) | -0.31 (-0.36 to -0.26) |
| Low-middle SDI | -0.02 (-0.03 to -0.01) | -0.17 (-0.20 to -0.14) | -5.61 (-6.42 to -4.78) | -0.25 (-0.30 to -0.20) |
| Low SDI | -0.03 (-0.03 to -0.03) | -0.12 (-0.14 to -0.11) | -4.26 (-4.38 to -4.14) | -0.55 (-0.57 to -0.52) |
| Andean Latin America | -0.01 (-0.01-0) | -0.17 (-0.19 to -0.15) | -2.66 (-3.77 to -1.53) | -0.17 (-0.19 to -0.15) |
| Australasia | 0 (-0.01-0) | -0.10 (-0.15 to -0.05) | -6.21 (-7.64 to -4.77) | -0.18 (-0.25 to -0.11) |
| Caribbean | 0 (-0.01-0) | -0.03 (-0.04 to -0.03) | -3.18 (-4.86 to -1.47) | -0.05 (-0.07 to -0.04) |
| Central Asia | -0.01 (-0.01 to -0.01) | -0.19 (-0.26 to -0.13) | -10.16 (-11.78 to -8.51) | -0.24 (-0.32 to -0.16) |
| Central Europe | -0.04 (-0.04 to -0.01) | -0.29 (-0.30 to -0.28) | -11.27 (-14.14 to -8.31) | -1.03 (-1.26 to -0.81) |
| Central Latin America | -0.01 (-0.01 to -0.01) | -0.08 (-0.09 to -0.07) | -10.10 (-12.51 to -7.62) | -0.44 (-0.54 to -0.33) |
| Central Sub-Saharan Africa | -0.01 (-0.01-0) | 0.02 (-0.03-0.08) | -3.75 (-4.06 to -3.44) | -0.03 (-0.1-0.03) |
| East Asia | -0.01 (-0.02-0) | -0.50 (-0.53 to -0.47) | -11.54 (-14.21 to -8.78) | -0.54 (-0.58 to -0.49) |
| Eastern Europe | 0 (-0.01-0.01) | -0.13 (-0.18 to -0.09) | -5.93 (-7.91 to -3.90) | -0.16 (-0.21 to -0.11) |
| Eastern Sub-Saharan Africa | -0.01 (-0.01 to -0.01) | -0.10 (-0.14 to -0.07) | -4.03 (-4.18 to -3.88) | -1.05 (-1.07 to -1.03) |
| High-income Asia Pacific | 0.20 (0.14-0.25) | -0.07 (-0.11 to -0.04) | -15.37 (-17.61 to -13.06) | -0.11 (-0.14 to -0.08) |
| High-income North America | 0.08 (0.05-0.11) | -0.07 (-0.10 to -0.04) | -4.11 (-5.32 to -2.88) | -0.19 (-0.24 to -0.14) |
| North Africa and Middle East | -0.02 (-0.03 to -0.02) | -0.16 (-0.18 to -0.13) | -5.71 (-6.15 to -5.26) | -0.18 (-0.20 to -0.15) |
| Oceania | 0 (0-0) | -0.08 (-0.10 to -0.06) | -1.11 (-1.23 to -0.98) | -0.08 (-0.10 to -0.06) |
| South Asia | 0.01 (0.01-0.01) | -0.09 (-0.17 to -0.02) | -19.09 (-21.18 to -16.94) | -0.16 (-0.27 to -0.06) |
| Southeast Asia | 0.02 (0.02-0.03) | -0.32 (-0.35 to -0.29) | -2.07 (-2.59 to -1.55) | -0.37 (-0.41 to -0.33) |
| Southern Latin America | -0.01 (-0.01-0) | -0.17 (-0.18 to -0.15) | -10.60 (-12.90 to -8.24) | -0.26 (-0.29 to -0.23) |
| Southern Sub-Saharan Africa | -0.01 (-0.01 to -0.01) | -0.05 (-0.05 to -0.04) | -1.72 (-1.98 to -1.46) | -0.07 (-0.08 to -0.07) |
| Tropical Latin America | 0 (0-0) | -0.06(-0.10 to -0.02) | -4.46 (-6.89 to -1.97) | -0.51 (-0.76 to -0.26) |
| Western Europe | 0.02 (0.01-0.02) | -0.13 (-0.14 to -0.11) | -6.90 (-8.62 to -5.13) | -0.48 (-0.58 to -0.37) |
| Western Sub-Saharan Africa | 0 (0-0) | -0.21 (-0.26 to -0.17) | -2.97 (-3.15 to -2.80) | -0.29 (-0.36 to -0.23) |

EAPC, estimated annual percentage change; ASIR, age-standardized incidence rate; ASPR, age-standardized prevalence rate; ASDR, age-standardized death rate; DALYs, disability-adjusted life years; SDI, socio-demographic index.
